# Progranulin inhibits LPS-induced macrophage M1 polarization via NF-кB and MAPK pathways

**DOI:** 10.1186/s12865-020-00355-y

**Published:** 2020-06-05

**Authors:** Lianlian Liu, Hongmei Guo, Aimei Song, Jiahui Huang, Yu Zhang, Shanshan Jin, Shutong Li, Liguo Zhang, Chengzhe Yang, Pishan Yang

**Affiliations:** 1 Department of Periodontology, School of Stomatology, Shandong University, 44 West Wenhua Road, Jinan, 250012 Shandong People’s Republic of China; 2 Shandong Provincial Key Laboratory of Oral Tissue Regeneration, Jinan, Shandong China; 3 Shandong Engineering Laboratory for Dental Materials and Oral Tissue Regeneration, Jinan, Shandong China; 4 Department of Oral and Maxillofacial Surgery, Qilu Hospital and Institute of Stomatology, Shandong University, Jinan, 250012 Shandong People’s Republic of China

**Keywords:** PGRN, Macrophage polarization, NF-κB, MAPK, Lipopolysaccharide (LPS)

## Abstract

**Background:**

Macrophage M1 polarization plays a pivotal role in inflammatory diseases. Progranulin (PGRN) has potential anti-inflammation action, however, the effect of PGRN on macrophage M1 polarization has been poorly studied. Our study aimed to investigate the effect of PGRN on lipopolysaccharide (LPS)-induced macrophage M1 polarization and clarify the underlying mechanisms.

**Methods:**

RAW264.7 cells were polarized to M1 macrophage by LPS with or without recombinant PGRN (rPGRN) and tumor necrosis factor alpha antibody (anti-TNF-α). A cell counting kit-8 assay (CCK-8), flow cytometry, Quantitative Real-Time PCR assay (q-PCR), Western blot assay and enzyme-linked immunosorbent assay (ELISA) were used to determine the effect of different treatments on cell proliferation, expression of surface phenotype marker and expressions and secretion of inflammatory cytokines. The activation of NF-κB/mitogen-activated protein kinase (MAPK) pathways and the nuclear translocation of NF-κB p65 were detected by Western blot and immunofluorescence respectively. THP-1 and primary bone marrow-derived monocytes (BMDMs) were also used to demonstrate effect of PGRN on expressions and secretion of inflammatory cytokines induced by LPS.

**Results:**

In RAW264.7 cells, rPGRN at concentrations below 80 ng/ml significantly promoted cell proliferation in dose dependent fashion. rPGRN significantly inhibited LPS-induced change of phenotype (CD86/CD206 ratio) and function (tumor necrosis factor (TNF-α) and inducible nitric oxide synthase (iNOS) expressions). LPS-stimulated secretion of TNF-α and activated phosphorylation of IKKα/β, IкBα, p65, JNK and p38 and the nucleus translocation of NF-кB p65 were also significantly downregulated by rPGRN. In addition, recombinant TNF-α (rTNF-α) significantly boosted TNF-α and iNOS expression vs the control group. Moreover, anti-TNF-α significantly inhibited LPS-induced TNF-α and iNOS expression. In THP-1 and BMDM cells, reversing effect of rPGRN on LPS-enhanced expressions of TNF-α and iNOS and secretion of TNF-α was further demonstrated.

**Conclusions:**

PGRN down-regulates LPS-induced macrophage M1 polarization in phenotype and function via NF-κB/MAPK signaling pathways.

## Background

Periodontitis is an infectious and inflammatory disease characterized by progressive infiltration of bacteria and inflammatory cytokines into periodontal tissues, resulting in attachment loss, alveolar bone absorption and apical migration of the junctional epithelium [1]. Although some special bacteria initiate periodontal inflammation, the host response motivated by bacterial products, for example, Porphyromonas gingivalis (P.g) LPS, plays an equal important role in mediating periodontal tissue breakdown [2]. Host-derived interleukin-1 (IL-1), IL-6, TNF-α, matrix metalloproteinases (MMPs) and prostanoids are main mediators for most of the tissue destruction [3–5].

The monocyte-macrophage plays an important role both in the adaptive immune response and innate immunity [6]. Plenty of evidences show that macrophages derived from circulating mononuclear cells and tissue resident cells exist in the diseased tissues of periodontitis and are leading players in immunoreaction against periodontal pathogens, contributing to the initiation of periodontal inflammation [7, 8]. The number of macrophages and macrophages-secreted pro-inflammatory cytokines including IL-1, IL-8, TNF-α and so on are elevated in periodontitis-associated gingival tissue biopsies [9]. Macrophages in gingival tissue play a dural role in the host’s defense against periodontal pathogen infection and in development of periodontitis depending on their polarization status [10].

When macrophages are recruited to diseased tissues, they are primed into different phenotypes depending on their exposure to different stimuli. When stimulated by LPS or/and interferon gamma (IFN-γ), macrophages differentiate into M1 phenotype, involved in pro-inflammatory activity and in host defense against bacteria and viruses. On the contrary, IL-4 or/and IL-13 induces macrophages to differentiate into M2 phenotype, behaving anti-inflammatory and pro-healing functions [7]. A variety of studies demonstrate that M1 macrophages predominate in gingival infiltrating macrophages of the mouse periodontitis model via P.g oral infection [11] and in human gingival tissue infected with periodontal disease [10, 12]. M1 macrophages produce a great mount of TNF-α, nitric oxide and IL-12 in response to P.g stimulation [13, 14]. Moreover, increased M1/M2 macrophage ratio augments orthodontic root resorption [15]. Therefore, modulating macrophage polarization status may be an important strategy for periodontal disease therapy [12].

PGRN is known as protein with the molecular mass of about 68.5 <kDa [16] which contains seven and one-half copies of granulin repeats [17]. As a multifunctional growth factor [18], PGRN is proved to be associated with tumorigenesis [19], neurodegeneration [20], wound healing [21] and early embryogenesis [22]. In regard of inflammation modulation, PGRN has been shown to promote proliferation of Treg cells and IL-10 secretion, and inhibit neutrophil degranulation, at least partly, through directly binding to TNF receptors (TNFRs) and antagonizing TNF-mediated pro-inflammatory signaling pathway [23, 24]. Additionally, PGRN has been demonstrated to have the protective role in osteoarthritis [23, 25], inflammatory bowel disease [26], psoriasis [27], and various autoimmune diseases [28, 29]. Our previous studies demonstrate that PGRN is highly expressed in periodontitis tissues such as the gingiva and gingival crevicular fluid and recombinant PGRN plays protective role in experimental periodontitis in rats [30] and promotes inflammatory periodontal bone defect regeneration in rats by inhibition of inflammation and osteoclast and promotion of osteogenesis [31]. However, the role of PGRN in modulating macrophage function has been seldom investigated [32]. Therefore, the current study was conducted to investigate the inhibition effect of PGRN on LPS-induced macrophage M1 polarization and the associated signaling pathways to provide a further insight of underling mechanism of PGRN anti-inflammatary activity.

## Results

### Effect of LPS on macrophage M1 polarization and PGRN expression

**Fig. 1:**
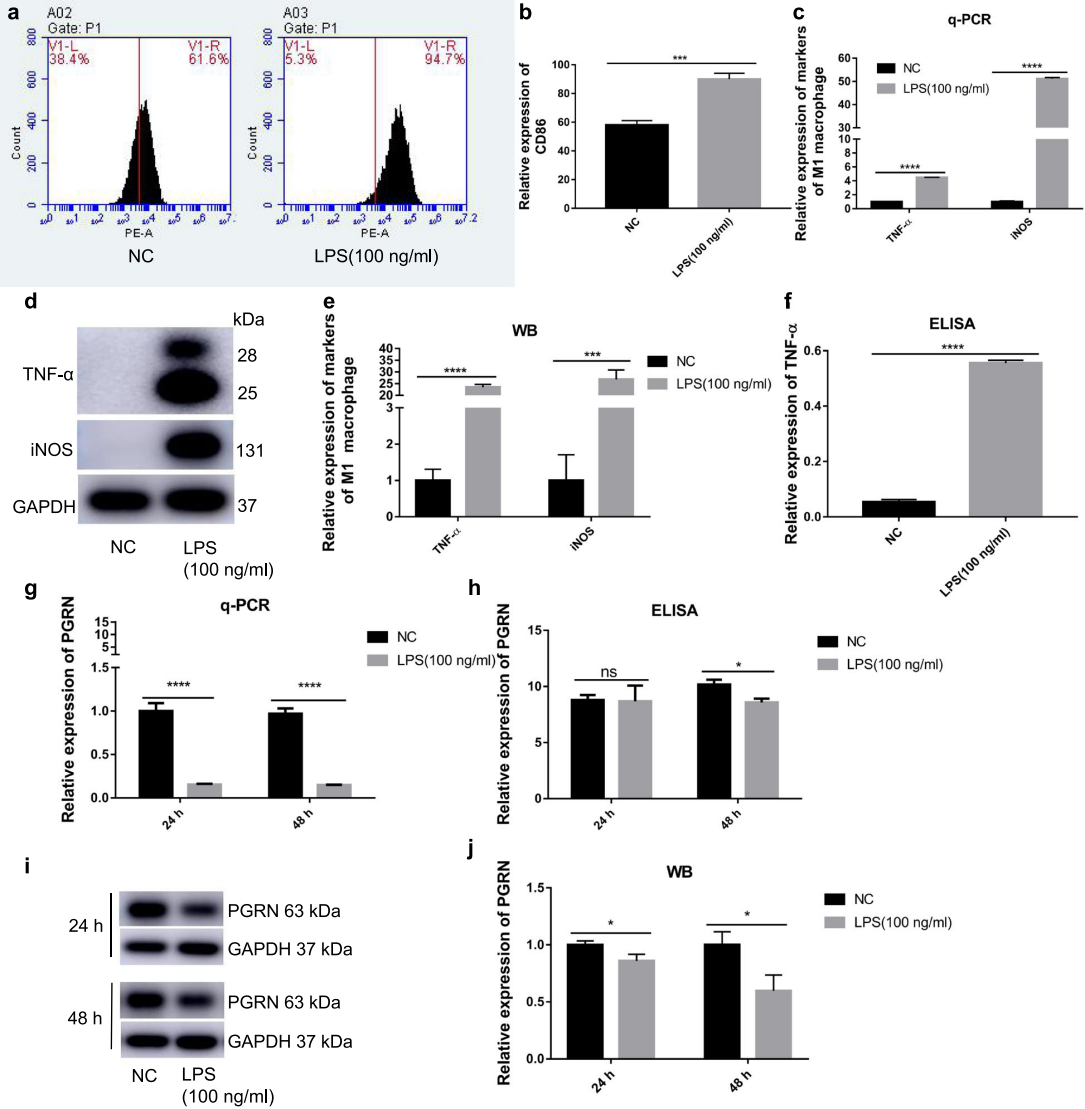
LPS induces macrophage M1 polarization whereas inhibits PGRN expression Cells were treated with 100 ng/ml LPS for 24 h and the normal medium containing equal amount of PBS to LPS groups was used as negative control. **a**, **b** CD86 expression were detected by flow cytometry. **c** The gene level of TNF-α and iNOS were determined with q-PCR. **d**, **e** The protein level of TNF-α and iNOS were revealed by western-blot. **f** The secretion level of TNF-α was evaluate by ELISA. Cells were treated with 100 ng/ml P.g-LPS for 24 and 48 h. **g** The gene level of PGRN were detected by q-PCR. **h** The secretion level of PGRN were evaluated by ELISA. **i**, **j** The protein level of PGRN were determined by Western blot. *N* = 3, *P* < 0.05 (*), *P* < 0.001 (***) or *p* < 0.0001(****)

After stimulated by 100 <ng/ml LPS or normal medium for 24 <h, LPS-stimulated RAW264.7 cells showed significantly higher expression of CD86, the special surface phenotype marker of M1, than negative control (Fig. 1a, b). In addition, mRNA and protein expression of iNOS and TNF-α, the special functional markers for M1, and secretion of TNF-α also significantly increased (Fig. 1c, d, e, f). Interestingly, compared to the control, LPS stimulation for 24 or 48 <h significantly down-regulated PGRN expression both in gene and protein expression (Fig. 1g, i, j) and its secretion was also significantly decreased after 48 <h stimulation (Fig. 1h). This suggests that PGRN may be involved in LPS-induced macrophage M1 polarization.

### Effects of rPGRN on proliferative capacity of RAW264.7 cells

**Fig. 2:**
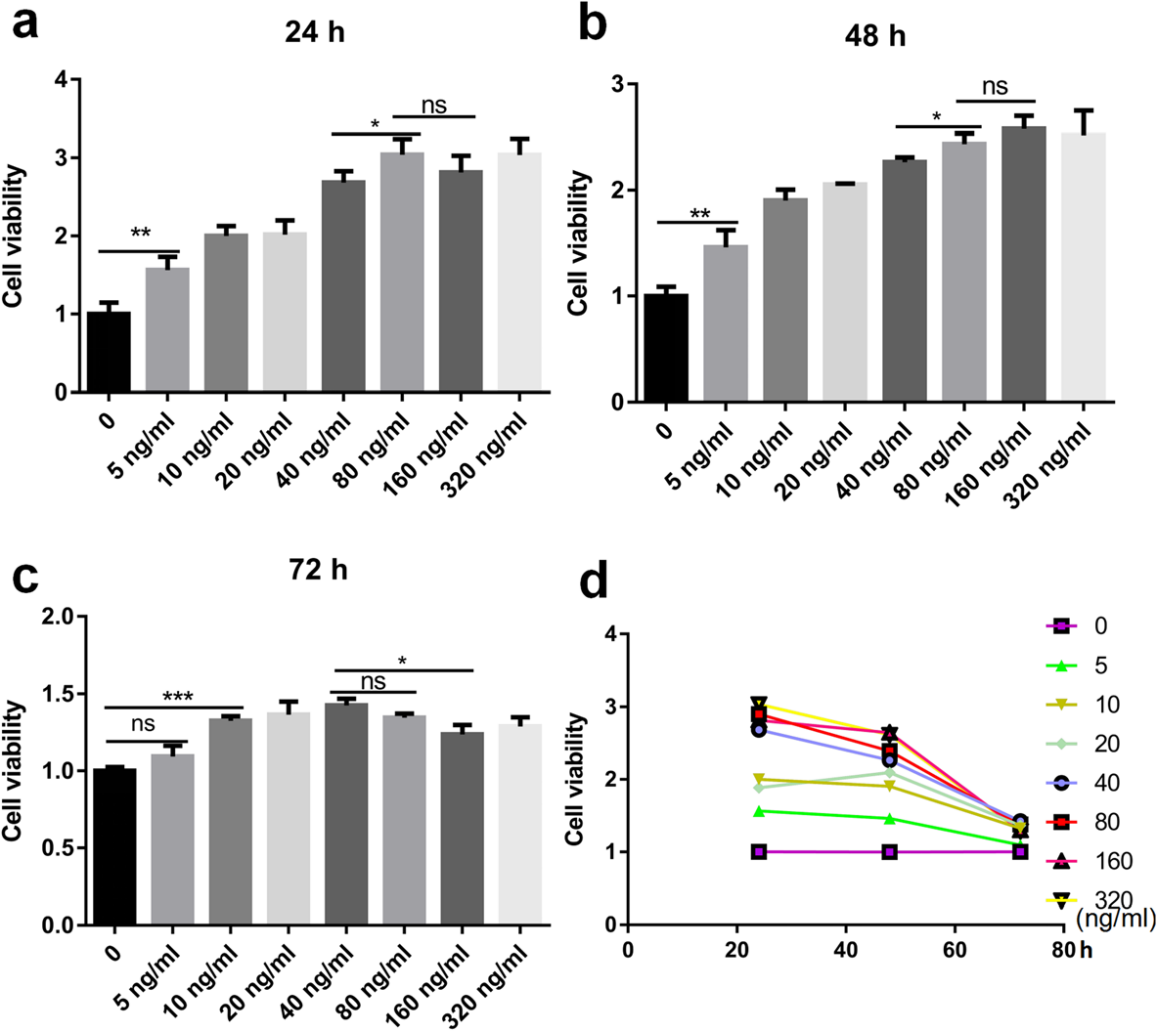
Effects of PGRN on RAW264.7 viability. **a**-**c** The effect of 5, 10, 20, 40, 80,160 and 320 ng/ml rPGRN on RAW264.7 cells was detected by CCK-8 assay after treatment for 24 h (**a**), 48 h (**b**) and 72 h (**c**). The optical density was normalized to a relative value of 100% for untreated cells. **d** Cell proliferation curve. *N* = 3, *P* < 0.05 (*), *P* < 0.01 (**) or *P* < 0.001 (***)

CCK-8 assays revealed that at concentrations below 80 <ng/ml, rPGRN significantly promoted cell proliferation in dose dependent fashion after cultured for 24 and 48 <h (Fig. 2a, b, d). Nevertheless, proliferative capacity of cells cultured at 160 and 320 <ng/ml rPGRN approached to flat, and even declined. Furthermore, the proliferative capacity of RAW264.7 cells treated with different concentrations of rPGRN was descending as processing time increases (Fig. 2a, b, c, d).

### Effects of rPGRN on LPS-induced M1 polarization in RAW264.7 cells

**Fig. 3:**
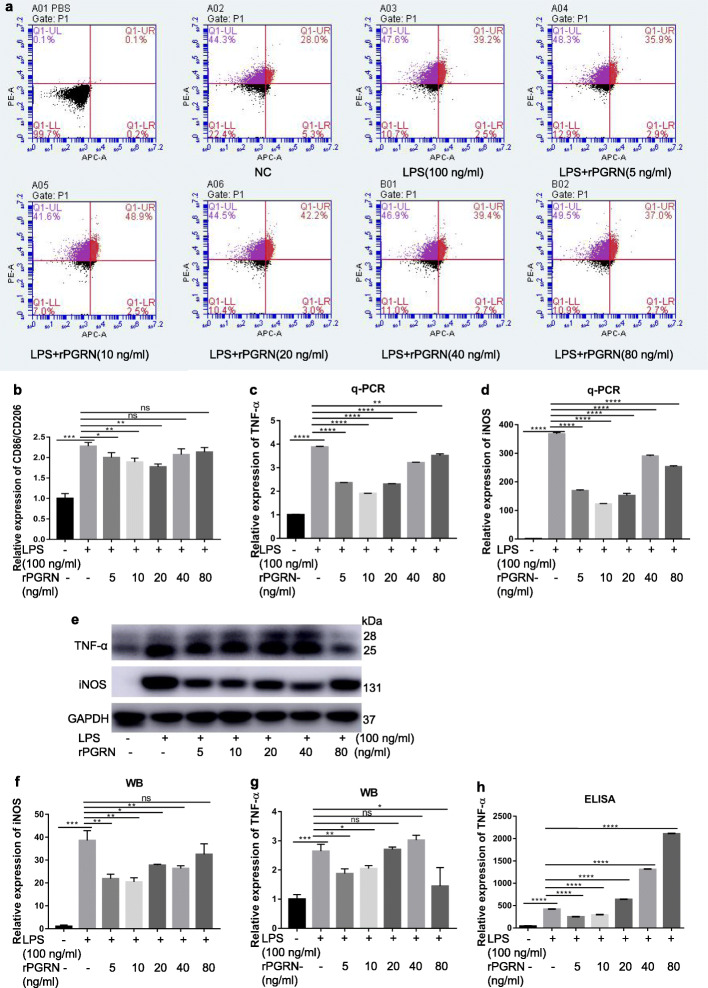
Inhibitory effects of rPGRN on CD86/CD206 ratio and inflammatory cytokines (TNF-α and iNOS) expression in LPS-stimulate RAW264.7 cells. Cells were treated with LPS plus 5, 10, 20, 40, and 80 ng/ml rPGRN for 24 h and normal medium containing equal amount of PBS to rPGRN and LPS groups was used as negative control. **a**, **b** CD86/CD206 ratio was detected by flow cytometry. **c**, **d** TNF-α (**c**) and iNOS (**d**) mRNA expression were determined with q-PCR. **e**-**g** TNF-α (**e**, **f**) and iNOS (**e**, **g**) protein expression were revealed by Western blot. **h** TNF-α secretion expression were evaluated by ELISA. *N* = <3, *P* < 0.05 (*), *P* < 0.01 (**), *P* < 0.001 (***) or *p* < 0.0001(****)

RAW264.7 cells were stimulated by LPS plus 0, 5, 10, 20, 40 and 80 <ng/ml rPGRN and normal medium containing equal amount of PBS to rPGRN and LPS groups was used as negative control. Their phenotype and function status were characterized by flow cytometry, q-PCR, Western blot and ELISA. The results showed that rPGRN at concentrations of 5, 10 and 20 <ng/ml significantly reversed LPS-promoted CD86/CD206 expression ratio, of which 20 <ng/ml rPGRN presented most obvious effect (Fig. 3a, b). Similarly, rPGRN significantly reversed LPS-enhanced mRNA of TNF-α and iNOS at concentrations from 5 to 80 <ng/ml, among which 10 <ng/ml rPGRN presented most obvious effect (Fig. 3c, d). It also inhibited LPS-enhanced protein expression of iNOS (at concentrations from 5 to 40 <ng/ml) (Fig. 3e, f) and TNF-α (at 5 and 10 <ng/ml) (Fig. 3e, g), as well as secretion of TNF-α (at 5 and 10 ng/ml) (Fig. 3h). This implies that PGRN can inhibit LPS-induced M1 polarization in RAW264.7 cells.

### The key role of TNF-α in LPS-induced macrophage M1 polarization

**Fig. 4:**
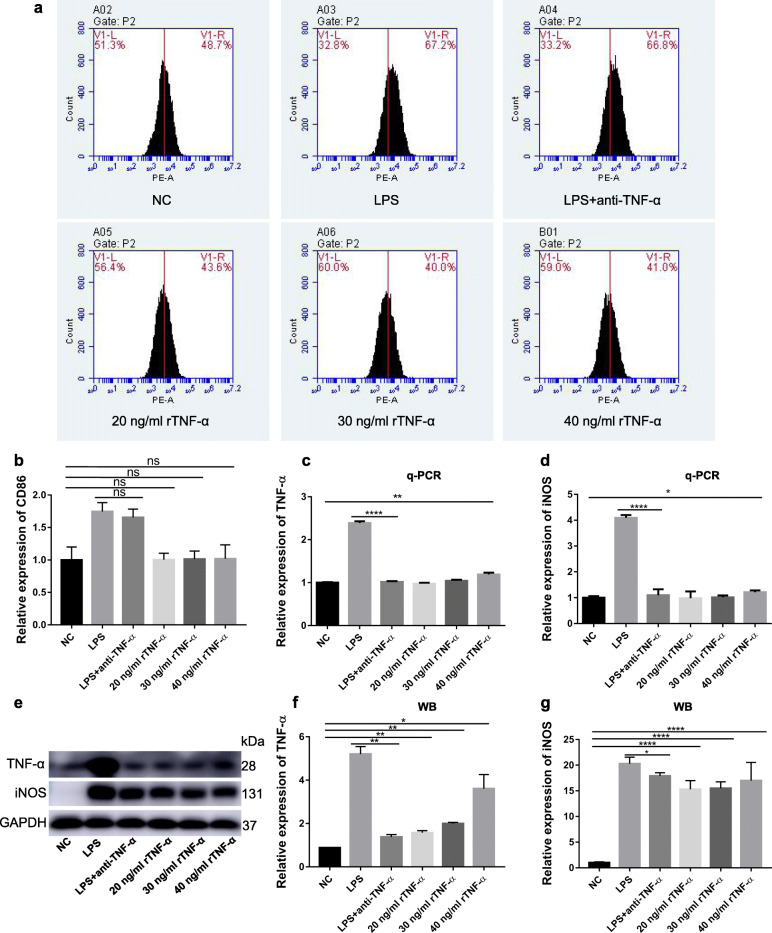
Effect of TNF-α on M1 polarization and of anti-TNF-α on LPS-induced M1 polarization in RAW264.7 cells. Cells were treated with LPS or plus anti-TNF-α or simple rTNF-α at concentration of 20, 30, 40 ng/ml for 24 h and normal medium containing equal amount of PBS to rTNF-α and LPS groups was used as negative control. **a**, **b** CD86 expression was detected by flow cytometry. **c**, **d** The gene level of TNF-α (**c**) and iNOS (**d**) were determined with q-PCR. **e**-**g** The protein level of TNF-α (**e**, **f**) and iNOS (**e**, **g**) were revealed by Western blot. *N* = 3, *P* < 0.05 (*), *P* < 0.01 (**) or *p* < 0.0001(****)

Previous research has identified that autocrine TNF-α plays a key role in apoptosis in LPS-induced macrophages [33]. It is also reported that PGRN exerts its anti-inflammatary action mainly via binding to TNFR1 to antagonize TNF-α pro-inflammation action [23]. To explore the key role of TNF-α and observe and conjecture whether PGRN anti-M1 polarization is related to TNFRs in LPS-induced macrophage M1 polarization, we stimulate RAW264.7 cells by rTNF-α, P.g-LPS and P.g-LPS plus anti-TNF-α. Results showed that no significant difference in CD86 expression existed between 20 to 40 ng/ml rTNF-α and the control groups and anti-TNF-α exerted no significant influence on P.g-LPS promoted CD86 expression (Fig. 4a, b). Nevertheless, 40 ng/ml rTNF-α significantly enhanced mRNA and protein expression of TNF-α and iNOS (Fig. 4c-g). More interestingly, anti-TNF-α significantly down-regulated LPS-stimulated expression of iNOS and intracellular TNF-α (Fig. 4c-g). This implies that secondary TNF-α expression followed by LPS stimulation plays a crucial role in LPS-activated M1 macrophage functional status.

### rPGRN inhibits LPS-activated NF-кB/MAPK pathways

**Fig. 5:**
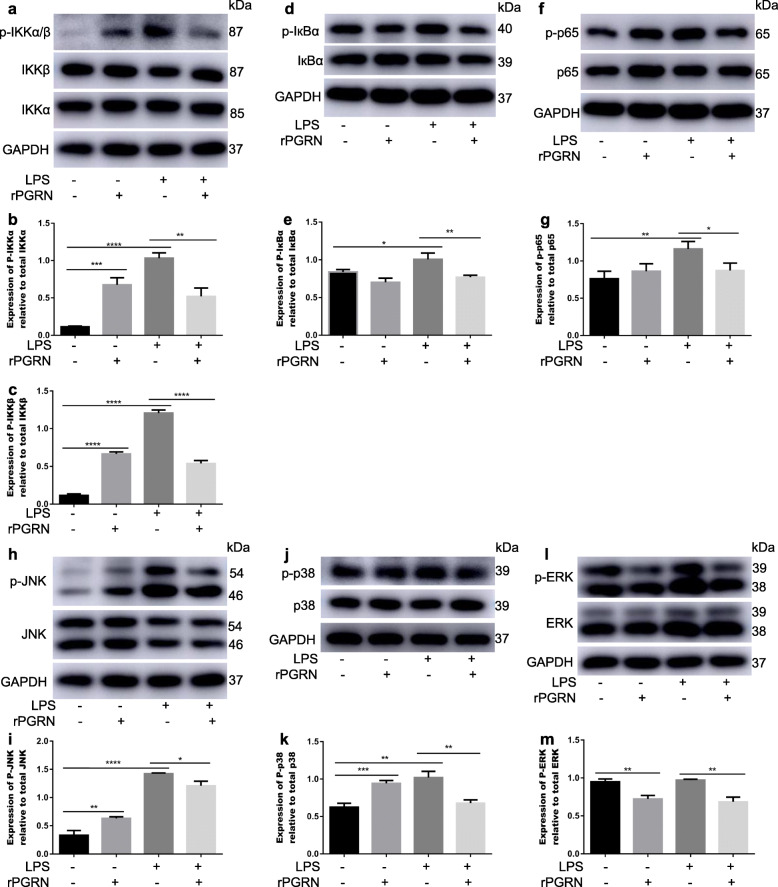
Effects of PGRN on LPS-induced activation of NF-κB and MAPK pathways. Cells were treated with rPGRN (10 ng/ml), LPS (100 ng/ml) and LPS + rPGRN for 60 min. **a**-**m** The protein level of IKKα/β (**a**-**c**), IкBα (**d**, **e**), p65 (**f**, **g**), JNK (**h**, **i**), p38 (**j**, **k**) and ERK (**l**, **m**) were determined with Western blot. *N* = 3, *P* < 0.05 (*), *P* < 0.01 (**), *P* < 0.001 (***) or *p* < 0.0001(****)

**Fig. 6:**
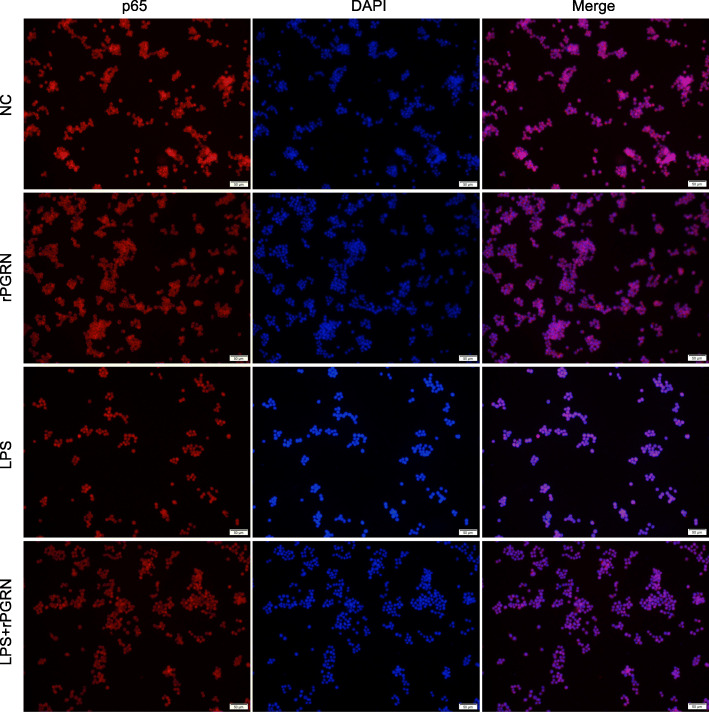
Effect of PGRN on nuclear translocation of NF-κB p65. Cells treated with rPGRN (10 ng/ml), LPS (100 ng/ml) and LPS + rPGRN for 60 min. The nuclear translocation of NF-κB p65 was evaluated by immunofluorescence staining (NF-κB p65, red fluorescent signals; DAPI, blue signals; magnification: × 200)

To clarify the mechanism of PGRN inhibition of LPS-induced M1 polarization in RAW264.7, the change of NF-кB and MAPK signaling molecules was detected by Western blot. As shown in Fig. 5, rPGRN significantly reduced LPS-induced phosphorylation of IKKα/β (Fig. 5a-c), IкBα (Fig. 5d, e), p65 (Fig. 5f, g), JNK (Fig. 5h, i) and p38 (Fig. 5j, k) and NF-кB p65 translocation from the cytoplasm to the nucleus (Fig. 6), though without LPS stimulation it was able of activating the phosphorylation of IKKα/β (Fig. 5a-c), JNK (Fig. 5h, i) and p38 (Fig. 5j, k) vs negative control group. In addition, rPGRN significantly down-regulated phosphorylation of ERK though LPS had no effect on it (Fig. 5l, m).

### rPGRN inhibits LPS-activated macrophage M1 polarization in BMDMs and THP-1 cells

**Fig. 7:**
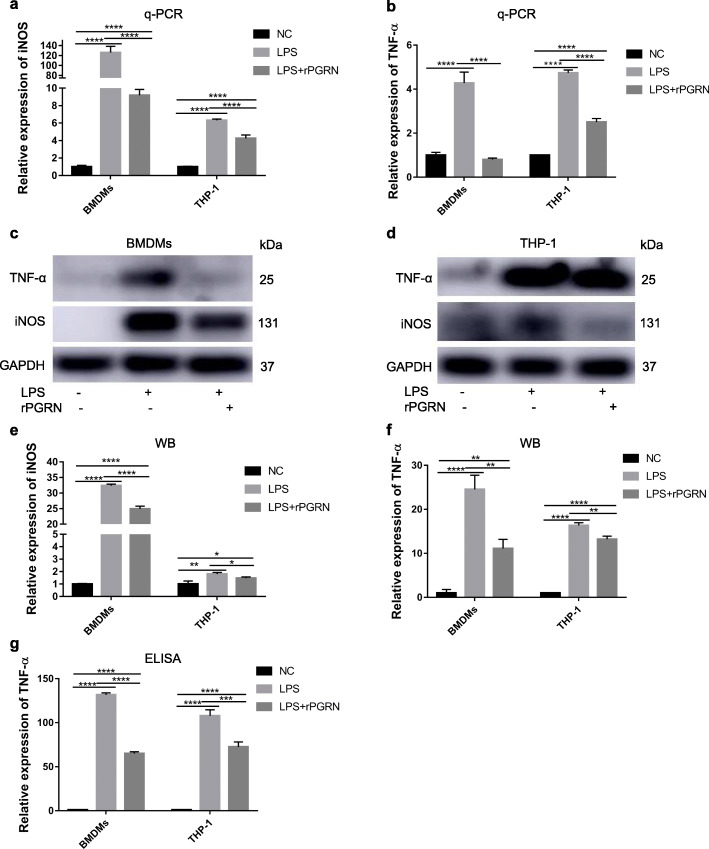
Inhibitory effects of PGRN on inflammatory cytokines (TNF-α and iNOS) expression in LPS-stimulate BMDMs and THP-1 cells. Cells were treated with LPS with or without 10 ng/ml rPGRN for 24 h and normal medium containing equal amount of PBS to rPGRN and LPS groups was used as negative control. **a**, **b** iNOS (**a**) and TNF-α (**b**) mRNA expressions were determined with q-PCR. **c**-**f** iNOS (**c**-**e**) and TNF-α (**c**, **d**, **f**) protein expressions were revealed by Western blot. **g** TNF-α secretion was evaluated by ELISA. *N* = 3, *P* < 0.05 (*), *P* < 0.01 (**), *P* < 0.001 (***) or *p* < 0.0001(****)

To further demonstrate above results in RAW.264.7 cells, BMDMs and THP-1 cell line were used to verify the effect of PGRN on functional change induced by LPS. As in RAW264.7 cells, rPGRN at 10 ng/ml significantly reversed LPS-enhanced mRNA and protein expression of TNF-α and iNOS (Fig. 7a-f) and secretion of TNF-α (Fig. 7g).

## Discussion

Plenty of studies indicate that macrophages, when as M1 phenotype, play a vital role in onset and development of periodontitis [34], while PGRN, identified as an endogenous antagonist of TNF-α by competitively binding to TNFRs, has potential positive action in autoimmune and inflammatory diseases, such as osteoarthritis [35] and periodontitis [30]. In this study, we demonstrated that rPGRN inhibited LPS-induced macrophage M1 polarization and these effects were associated with NF-кB and MAPK pathway inhibition.

Porphyromonas gingivalis is the main pathogen bacteria of periodontitis and P.g-LPS plays a key role in mediating differentiation and function of M1 macrophages [13, 36, 37] and in periodontal tissue breakdown [2]. However, other report demonstrate that exposure to P.g-LPS results in diminished pro-inflammatory cytokine production [38]. There is also study showing that there is not significant difference in macrophage polarization between periodontitis and healty tissues [39]. This suggests that, given the importance of macrophages in inflammatory diseases, the effect of P.g-LPS on macrophage polarization needs to be elucidated. The present study, as previously reported [13, 14, 36], validated that macrophages underwent M1 phenotypic and functional changes under P.g-LPS stimulation. Interestingly, with the increase of the expression of M1-specific marker and functional molecules, the expression of endogenous PGRN was significantly reduced, suggesting that PGRN is involved in the regulation of M1 polarization under P.g-LPS stimulation.

It is well documented that CD86 and CD206 are special phenotypic markers of M1 and M2 macrophages respectively and iNOS and TNF-α are special functional markers of M1. To further investigate the effect of PGRN on M1 polarization, RAW264.7 cells were stimulated by P.g-LPS with or without rPGRN. We found that rPGRN at range of 5 to 20 ng/ml suppressed LPS-enhanced CD86/CD206 ratio and the expression of TNF-α and iNOS. This demonstrates that PGRN can reverse macrophage M1 polarization under P.g-LPS stimulation, in consistent with study by Yoo et al., showing that PGRN reduces inflammatory gene expression in palmitate-induced macrophage [40].

It has been reported that PGRN acts via competitively antagonizing TNFRs, while TNF-α was one of the most important marker molecules of M1 macrophages [41]. In order to clarify the role of secondary TNF-α in M1 polarization and to speculate whether PGRN anti-M1 polarization is related to TNFRs, we stimulated RAW264.7 with exogenous TNF-α and found that TNF-α enhanced expression of iNOS and endogenous TNF-α, but exerted no influence on special surface phenotype marker CD86 expression. More important, anti-TNF-α treatment down-regulated P.g-LPS promoted expression of iNOS and intracellular TNF-α. This implies that secondary TNF-α plays an important role in LPS activated M1 polarization. Considering the key role of TNFR1 in PGRN antagonizing the TNF-mediated inflammatory signaling pathway, it is conjectured that PGRN-reversed macrophage M1 polarization challenged by P.g-LPS may be associated with blockage of TNFR1.

Based on its role in up-regulating the expression of pro-inflammatory genes, NF-κB pathway has widely been considered as a classical pro-inflammatory signaling pathway [42]. When exposed to stimulus such as LPS or TNF-α, inhibitors of p65/p50 heterodimer (IκBs) is phosphorylated and degraded by IKKs, which result in p65/p50 neuclear translocation. Eventually, the transcription of target genes is activated [43]. To explore if NF-κB pathway is involved in reversing action of PGRN for LPS-promoted M1 polarization, RAW264.7 cells were treated by rPGRN (10 ng/ml) and P.g-LPS with or without rPGRN. Our results verified that presence of rPGRN suppressed activation of NF-кB induced by LPS, decreased phosphorylation of IкB kinase (IKKα/β), and IкBα, and reduced nuclear translocation of NF-кB p65 and its phosphorylation. In addition, given that MAPK pathway is also critical pro-inflammatory signaling pathway [44–46] and especially JNK and p38 are widely considered motivators of IкBα degration, we also examined whether anti-M1 polarization mechanism of PGRN may be associated with MAPK pathway. As shown in Fig. 6, 10 ng/ml rPGRN significantly inhibited LPS-activated JNK and p38, though PGRN moderately phosphorylate JNK and p38. These results suggest that NF-кB and MAPK/ JNK/ p38 pathways are involved in reversing action of PGRN for LPS-promoted M1 polarization.

## Conclusions

Our study demonstrates that P.g-LPS stimulates M1 polarization via NF-κB and MAPK pathways and TNF-α mediates, while PGRN efficiently inhibits this process. However, the mechanism and specific role of TNFRs in PGRN mediated inhibition of M1 polarization remains to be explored. Besides, though we have shown that PGRN protects against experimental periodontitis [30] and promotes inflammatory periodontal bone defect regeneration in rats [31], whether this in vivo efficacy of PGRN is related to anti-M1 polarization waits for investigation.

## Methods

### Cell culture and polarization stimulation

RAW264.7 cells were obtained from Stem Cell Bank, Chinese Academy of Sciences. Mouse BMDMs were isolated from femur and tibia of C57BL/6 mice which were purchased from Institute of Shandong University Animal Experimental Center and differentiated into M0 macrophage by 25 ng/ml recombinant macrophage colony-stimulating factor (M-CSF) (R&D Systems, Minneapolis, MN, USA) treatment for 4 days. THP-1 cells (Stem Cell Bank, Chinese Academy of Sciences) were differentiated into M0 by 100 ng/ml PMA (Sigma, USA) treatment for 48 h. All cells were cultured in DMEM (Hyclone, Logan, UT, USA) containing 10% foetal bovine serum (FBS) (BioInd, Kibbutz, Israel) at 37 °C with 5% CO_2_. When reached 80% confluence, cells were scraped, dissociated and counted and then plated in 6-well plates at a concentration of 2 × 10^5^/ml (in RAW264.7 cells) or 2 × 10^6/^ml(in BMDMs and THP-1 cells). When reaching 60% confluence, cells were stimulated by 100 ng/ml P.g-LPS (InvivoGen, San Diego, CA, USA) with or without different doses of rPGRN (Sino Biological, Beijing, China) and TNF-α antibody (5 μg/ml; Abcam, Cambridge, UK) or a variety of concentrations of rTNF-α (Peprotech, Rocky Hill, NJ, USA) for 24 or 48 h, depending on different experimental goals. The normal medium containing equal amount of PBS to rPGRN and LPS groups was used as negative control.

### Flow cytometry

The surface markers of stimulated cells were detected by flow cytometry. Briefly, RAW264.7 cells were collected from 6-well plates after stimulation for 24 h and washed three times with PBS. The cell suspension respectively containing 1 × 10^6^ M0-unpolarized or M1-polarized cells was then divided into 1.5 ml EP tubes and incubated with blocking antibody CD16/32 (Biolegend, San Diego, CA, USA) on ice for 10 min. After washed twice, cells were incubated in PBS plus Intrapore Permeabilization reagent and the following antibodies (PE anti-mouse CD86 and APC anti-mouse CD206 (both from Biolegend)) on ice for 30 min in the dark. After washed twice, cells was suspended in 500 μl PBS with 3% FBS and then detected by flow cytometry (BD Biosciences, San Diego, CA, USA).

### Cell proliferation assay

RAW264.7 cells were counted and seeded in a 96-well plate (3000/well) and cultured in medium plus rPGRN at variable concentrations (5, 10, 20, 40, 80, 160, 320 ng/ml) or normal medium for 24, 48 and 72 h. Then, according to the instruction, the culture medium was replaced by 100 μl DMEM medium plus 10 μl cck-8 reagent (MCE, Shanghai, China) and the cells were incubated for another 2 h at 37 °C in 5% CO_2_ incubator. Absorbance at 450 nm was read by a microplate reader (SPECTRO star Nano) and cell viability was verified by the percent of the absorbance of various concentrations versus control group.

### Quantitative real-time PCR

Real-time reverse transcriptional polymerase chain reaction was performed as follows. Total cellular RNA was isolated from RAW264.7, THP-1 and BMDM cells with TRIzol reagent (Takara, Kusatsu, Japan) and then reverse-transcribed into cDNA with PrimeScript® RT reagent kit with gDNA Eraser (Takara) according to the concentration. Afterwards, Real-time PCR was performed with SYBR® Premix Ex Taq™ II (Takara). Analysis was performed on Light Cycler 96 Real-Time PCR System (Roche, Basel, Switzerland). The housekeeping gene GAPDH was used for normalization. The primers used in this study are shown in Table 1.

**Table 1:** primer sequences used in this study

Gene	Full name	Forward (5′-3′)	Reverse (5′-3′)
TNF-α iNOS PGRN	Tumor necrosis factor-α Inducible nitric oxide synthase Progranulin	GCCTCTTCTCATTCCTGCTTG TGGAGCCAGTTGTGGATTGTC CCTGGTTCACACACGATGCG	GGCCATTTGGGAACTTCTCA GGTCGTAATGTCCAGGAAGTAG CAGGTGGTCGGAACAGCAGA

### Western blot assay

Cells were lysed with radioimmunoprecipitation assay (RIPA) lysis buffer (Solarbio, Beijing, China) on ice for 30 min and decomposed with ultrasonic. Afterwards, the mixture was centrifugated at 12,000 rpm at 4 °C for 15 min to eliminate the dead cell debris. Protein concentration was detected by BCA protein assay kit (KeyGEN BioTECH, Nanjing, China). After denaturation with loading buffer at 100 °C, 20 μg protein samples were separated in 10% sodium dodecyl sulfate-polyacrylamide gel electrophoresis (SDS-PAGE) and then transferred to polyvinylidene fluoride (PVDF) membranes (Millipore, Billerica, MA, USA). Membranes were blocked in 5% milk for 1 h, then covered with the primary antibodies overnight at 4 °C and incubated with anti-mouse or anti-rabbit secondary antibodies (1:10000; Proteintech, Chicago, IN, USA) for 1 h at room temperature on shaker. The protein bands were visualized with chemiluminescent HRP reagents (Millipore, Darmstadt, Germany). Image J 1.44 was used to analyze the protein expression. The primary antibodies were as follows: TNF-α (1:1000; CST, Danvers, MA, USA), iNOS (1:1000; Abcam, Cambridge, UK), Arg-1 (1:500; Santa Cruz, CA, USA), PGRN (1:1000; Abcam), NF-кB p65 (1:1000; CST), phospho-NF-кB p65 (1:1000; CST), IкBα (1:1000; CST), phospho-IкBα (1:1000; CST), IKKα (1:500; Santa Cruz), rabbit anti-IKKβ (1:1000; CST), phospho-IKKα/β (1:1000; CST), p38 (1:1000; Abcam), phospho-p38 (1:1000; Abcam), JNK (1:1000; Abcam), phospho-JNK (1:1000; Abcam), ERK1/2 (1:1000; Abcam), phospho-ERK1/2 (1:1000; Abcam), GAPDH (1:10000; Proteintech).

### Enzyme-linked immunosorbent assay (ELISA)

Cell supernatant was collected from RAW264.7, THP-1 and BMDM cells, centrifuged at 12,000 rpm at 4 °C for 10 min and the concentrations of TNF-α and PGRN were measured with ELISA kits (Novus Biologicals, CO, USA or Abcam, Cambridge, UK). All samples were assayed in triplicate and measured at 450 nm wavelength.

### Immunocytochemistry

RAW264.7 cells plated in 24-wells plate were fixed with 4% paraformaldehyde for 10 min in a fume hood, permeabilized using 0.5% Triton X-100 (Solarbio, Beijing, China) for 10 min and rinsed three times with cold PBS for 5 min for each. After being blocked with 5% goat serum for 1 h, cells were incubated with an anti-NF-κB p65 primary antibody (1:500; CST) overnight at 4 °C. After washed with cold PBS twice, 1‰ Tween PBS once and incubated with Alexa Fluor 594-conjugated goat anti-rabbit IgG secondary antibody (1:500; Proteintech) for 1 h in the dark, nuclei were stained with DAPI (Proteintech). Images were detected with fluorescence microscope (OLYMPUS, Tokyo, Japan).

### Statistical analysis

Experiment results were expressed as the mean ± SD of at least 3 independent experiments and GraphPad Prism 7 software (San Diego, CA) was used for statistical analysis. Difference between groups was assessed by Unpaired two-tailed Student’s *t-*test and one-way ANOVA. Statistical significance was expressed as *P* < 0.05 (*), *P* < 0.01 (**), *P* < 0.001 (***) or *p* < 0.0001(****).

## Data Availability

The datasets in the current study are included in the published article or available from the corresponding author on reasonable request.

## Abbreviations

PGRN: Progranulin
rPGRN: Recombinant PGRN
LPS: Lipopolysaccharide
IFN-γ: Interferon gamma
TNF-α: Tumor necrosis factor
iNOS: Inducible nitric oxide synthase
CCK-8: Cell counting kit-8
P.g: Porphyromonas gingivalis
IL-1/4/6/8/10//12/13: Interleukin-1/4/6/8/10/12/13
TNFRs: Tumor-necrosis-factor receptors
MMPs: Matrix metalloproteinases
MIP-1α: Macrophage inflammatory protein
rPGRN: Recombinant PGRN
rTNF-α: Recombinant TNF-α
anti-TNF-α: Tumor necrosis factor alpha antibody
MAPK: Mitogen-activated protein kinase
q-PCR: Quantitative Real-Time PCR
ELISA: Enzyme-linked immunosorbent assay
Fig.: Figure
